# Supplementary material for: Interplay between metavalent bonds and dopant orbitals enables the design of SnTe thermoelectrics
Source: Nat Commun. 2024 Oct 23;15:9133. doi: 10.1038/s41467-024-53599-2 (PMC11500016; doi:10.1038/s41467-024-53599-2)
Supplement: Supplementary file 1 — Supplementary Information [file 41467_2024_53599_MOESM1_ESM.pdf]

Supporting Information

Interplay between metavalent bonds and dopant orbitals enables the design of SnTe thermoelectrics

Guodong Tang[1, #, *], Yuqi Liu[1, #], Xiaoyu Yang[2, #], Yongsheng Zhang[3], Pengfei Nan[2], Pan Ying[1], Yaru Gong[1], Xuemei Zhang[4, 5, *], Binghui Ge[2], Nan Lin[6], Xuefei Miao[1], Kun Song[7], Carl-Friedrich Schön[6], Matteo Cagnoni[8], Dasol Kim[6], Yuan Yu[6, *], Matthias Wuttig[6, 9, *]

[1] National Key Laboratory of Advanced Casting Technologies
MIIT Key Laboratory of Advanced Metallic and Intermetallic Materials Technology
Engineering Research Center of Materials Behavior and Design, Ministry of Education
Nanjing University of Science and Technology, Nanjing, 210094, China

[2] Key Laboratory of Structure and Functional Regulation of Hybrid Materials of Ministry of Education
Institutes of Physical Science and Information Technology
Anhui University, Hefei 230601, China

[3] Advanced Research Institute of Multidisciplinary Sciences
Qufu Normal University
Qufu, Shandong Province, 273165, China

[4] School of Physics and Electronic Information Engineering, Engineering Research Center of Nanostructure and Functional Materials, Ningxia Normal University
Guyuan, Ningxia 756000, China

[5] Science Island Branch of Graduate School, University of Science and Technology of China
Hefei 230026, China

[6] Institute of Physics (IA)
RWTH Aachen University
52056 Aachen, Germany

[7] School of Mechanical and Power Engineering
Nanjing Tech University
30 Puzhu South Road, Nanjing Jiangsu, China

[8] Department of Electronics and Telecommunications
Politechnico di Torino
Corso Duca degli Abruzzi 24, 10129 Torino, Italy

[9] Peter Grünberg Institute—JARA-Institute Energy-Efficient Information Technology (PGI-10)
Forschungszentrum Jülich GmbH
Jülich 52428, Germany

[#] These authors contributed equally to this work
[*] To whom correspondence should be addressed
E-mail: tangguodong@njust.edu.cn
E-mail: saxuemei@mail.ustc.edu.cn
E-mail: yu@physik.rwth-aachen.de
E-mail: wuttig@physik.rwth-aachen.de

Keywords: SnTe; thermoelectric; effective mass; optimum carrier concentration; metavalent bonding

Lorenz number calculations in detail:

In general, the total (κ) consists of the electronic thermal conductivity (κ_e) and lattice thermal conductivity (κ_L). The electronic part κ_e is directly proportional to the electrical conductivity σ through the Wiedemann-Franz relation, $\kappa_e = L\sigma T$, where L is Lorentz number and its value is calculated by the SPB model. The Lorenz number can be given as[1, 2]:

$$L = \left(\frac{k_B}{e}\right)\left(\frac{(r+7/2)F_{r+5/2}(\eta)}{(r+3/2)F_{r+1/2}(\eta)} - \left[\frac{(r+5/2)F_{r+3/2}(\eta)}{(r+3/2)F_{r+1/2}(\eta)}\right]^2\right) \quad (1)$$

For the Lorenz number calculation, we should get reduced Fermi energy η first; the calculation of η can be derived from the measured Seebeck coefficients by using the following relationship:

$$S = \pm\frac{k_B}{e}\left(\frac{(r+5/2)F_{r+3/2}(\eta)}{(r+3/2)F_{r+1/2}(\eta)} - \eta\right) \quad (2)$$

where $F_n(\eta)$ is the n−th order Fermi integral,

$$F_n(\eta) = \int_0^\infty \frac{\chi^n}{1+e^{\chi-\eta}} d\chi \quad (3)$$

where e is the electron charge, k_B is the Boltzmann constant, h is the Planck constant, and r is the scattering factor. The scattering factor (r) is -1/2 since acoustic phonon scattering has been assumed as the main carrier scattering mechanism near room temperature (RT). Lorentz number can be obtained by combining equations (1), (2) and (3).

Modified Williamson-Hall method to calculate dislocation density in detail：

Instrumental broadening is calibrated based on the values of FWHM of the standard reference material 1976 b. Taking into account that size and strain broadening are diffraction order independent and dependent, respectively, Williamson and Hall suggested that the FWHM of line profiles can be written as[3]:

$$\Delta K = 0.9/\mathrm{d} + \Delta K^{d} \tag{4}$$

where ΔK^{d} is the strain contribution to line broadening and d is the volume averaged grain size or particle size. and g = K at the exact Bragg position. When strain is caused by dislocations, ΔK^{d} has the following form[4-6]:

$$\Delta K^{d} = A(\rho^{*})^{1/2} + A'(\rho^{*})^{1/2} \tag{5}$$

where A and A' are parameters determined by the effective outer cutoff radius of dislocations, R_e, and the auxiliary parameters R_1 and R_2, respectively. N_D^* and Q^* are the formal values of dislocation density and the correlation factors, respectively, they are related to the true values N_D^* and Q^* as[7]:

$$N_D^{*} = N_D\,(\pi g^2 b^2 \bar{C})/2 \tag{6}$$

$$Q^{*} = Q\,(\pi g^2 b^2 \bar{C})^2/4 \tag{7}$$

where b is the magnitude of Burgers vector and $\bar{C}$ is the average dislocation contrast factor for a particular reflection g and g=K at the exact Bragg peak position.

Equation (8) shows that if dislocations are the primary source of strain in a crystal, the proper scaling factor of the breadths (or FWHM) of line profiles is ($K\bar{C}^{1/2}$) instead of merely K. Thus, equation (4) will be written as:

$$\Delta K = 0.9/d + (\pi A^2 b^2)^{1/2} N_D^{1/2} (K\bar{C}^{1/2}) + O(K\bar{C}^{1/2})^2 \tag{8}$$

where $O=(\pi A'b^2/2)\,Q^{1/2}$.

The full widths at half maximum (FWHM) of the diffraction profiles are determined as the widths of Gaussian functions fitted to the experimental diffraction data. According to the XRD diffraction peak broadening effect, the MWH method for calculating dislocation density derived from Ungár and Borbély can be written as follows[7, 8]. The derivation of the equation is shown in Supporting Information.

$$\Delta K=0.9/d+(\pi A^2b^2)^{1/2}N_D^{1/2}(K\bar{C}^{1/2})+O(K\bar{C}^{1/2})^2 \quad (9)$$

where d and K represent the apparent size parameters and the diffraction vector (K=2sinθ/λ, ΔK = 2cosθΔθ/λ, θ and λ are the Bragg angle and the wavelength of X-rays), A is constant depending on the outer cut-off radius of dislocations, b is the magnitude of Burgers vector and N_D is dislocation density and O is a higher order term of $K\bar{C}^{1/2}$ which is often ignored. The average contrast factor is a linear function of the fourth-order invariant of the Miller (hkl) indices of the different reflections[9]:

$$\bar{C}=\bar{C}_{h00}\,(1-q\mathrm{H}^2) \quad (10)$$

where $H^2=(h^2k^2+h^2l^2+k^2l^2)/(h^2+k^2+l^2)^2$ and $\bar{C}_{h00}$ (Table S7) is the average dislocation contrast factor corresponding to the $h00$ reflection determined by elastic modulus[9]. In a polycrystalline cubic metal, the $\bar{C}$ is a constant depending on the anisotropic elastic constants C_{11}, C_{12} and C_{44} obtained by equation (10)[10]. Four diffraction angles are selected for K ~ ΔK plot (Figure S23a), and corresponding indices of the crystal plane and FWHM are listed in Table S8. The value of 0.9/d is obtained by linear fitting. The value of q is obtained by fitting the formula of $(\Delta K-0.9/d)^2/K^2 - H^2$ as follows[11]:

$$\frac{(\Delta K-0.9/d)^2}{K^2}=(\pi A^2b^2/2)N_D\bar{C}_{h00}(1-qH^2) \quad (11)$$

where the calculated q equals −6.86. Finally, the dislocation density can be determined by the best linear fit between $\Delta K \sim KC^{1/2}$ plot in equation (8) (Figure S23b).

The lattice thermal conductivity calculated by the Klemens model in detail：

According to the phonon scattering model developed by Debye-Klemens, the lattice thermal conductivity can be expressed as

$$\kappa_{lat} = \frac{k_B}{2\pi^2 \upsilon}\left(\frac{k_B T}{\hbar}\right)^3 \int_0^{\Theta_D/T} \tau_{tot}(x) \frac{x^4 e^x}{(e^x-1)^2} dx \quad (12)$$

where υ is the average phonon velocity, $\hbar$ is the reduced Planck's constant, Θ_D is the Debye temperature, $x \equiv \hbar\omega/k_B T$, where ω is the phonon angular frequency. The total relaxation time, τ_{tot}, can be calculated according to Matthiessen's rule including the primary scattering terms from the Umklapp process (τ_U^{-1}), point defects (τ_{PD}^{-1}), and dislocation cores and strain fields (τ_{Dis}^{-1}) for materials studied in this work.

The relaxation time associated with Umklapp (τ_U) and Normal (τ_N) phonon-phonon scattering can be obtained from

$$\tau_U^{-1} + \tau_N^{-1} = A_N \frac{2}{(6\pi^2)^{1/3}} \frac{k_B \overline{V}^{1/3} \gamma^2 \omega^2 T}{\overline{M} v^3} \quad (13)$$

where A_N is an additional factor (=1.0 in this case) for integrating (τ_U) and (τ_N), γ is the Grüneisen parameter, $\overline{M}$ is the average atomic mass, v is the average speed of sound, and $\overline{V}$ is the average atomic volume.

The relaxation time associated with the point defect scattering is given by

$$\tau_{PD}^{-1} = \frac{\overline{V}\omega^4}{4\pi v^3}\Gamma \quad (14)$$

$$\Gamma_i = x_i\{(\Delta M_i/M)^2 + \epsilon[\Delta\delta/\delta]^2\} \quad (15)$$

$$\Gamma = \Sigma_i \Gamma_i \quad (16)$$

where x_i is the fractional concentration of component i of the alloy. ΔM_i is the mass difference between the impurity and host atom, M is the atomic mass of the matrix, $\Delta\delta$ is the atomic radius difference, δ is the atomic radius of the matrix atom, and ϵ is a phenomenological parameter.

The phonon relaxation time associated with the scattering of dislocation cores and strains can be expressed as:[12]

$$\tau_{DC}^{-1} = N_D \frac{\bar{V}^{4/3}}{v^2} \omega^3 \quad (17)$$

$$\tau_{DS}^{-1} = \frac{1}{3} N_D b^2 \gamma^2 [\ln(R/b)]^2 \omega \quad (18)$$

where N_D is the dislocation density, b is the Burgers vector, and R is the range of the strain field of dislocations, which equals the grain size (10 μm).

Energy conversion efficiency and output power of single-leg TE device for $Sn_{0.8}Al_{0.08}Sb_{0.15}Te$-4%$AgBiTe_2$.

Here, we designed the single-leg TE device for $Sn_{0.8}Al_{0.08}Sb_{0.15}Te$-4%$AgBiTe_2$ and simulated its output power P_{out} and energy conversion efficiency based on COMSOL Multiphysics (Figure S20)[13, 14]. Figure S20 shows the temperature distribution and corresponding electric potential distribution of the single-leg TE device. Conversion efficiency can be given by the following equation according to its definition:

$$P_{out} = IV_L = (V_{OC} - IR) \tag{19}$$

$$\eta = \frac{\mathrm{Pout}}{\mathrm{Qh}} \times 100\% \tag{20}$$

where P_{out} is the output power, Q_h is the heat flow into the hot side, I is the current in the circuit, V_L is the load voltage, V_{OC} is the open-circuit voltage, and R is the internal resistance. Corresponding simulation parameters are shown in the Figure S20d. One can see that the simulated results (the red line) matched well with calculated results (the blue line) using equation (20) and the maximum thermoelectric conversion efficiency η_{max}= 16.7% can be reached (at T_c=300 K and T_h=873 K).

Table S1. Thermoelectric transport properties of various dopants in SnTe-based materials. Seebeck coefficient (*S*) values at 300 K and maximum Seebeck coefficient, maximum power factor (*PF*), band effect, band convergence (BC) and resonant level (RL), electronic structure (band gap calculated by DFT and energy offset between the light-hole band and heavy-hole band).

Dopants	S_{300K} (μV K^{-1})	S_{max} (μV K^{-1})	PF (μWcm^{-1} K^{-2})	Band Effect	Band Gap (eV)	ΔE(L-Σ) (eV)	Ref
SnTe	24	110	15.7	/	0.06	0.35	This work
Ca	48	186	26	BC	0.07	0.2	[15]
Mg	38	200	30.3	BC	0.26	0.18	[16]
Mn	60	275	15.5	BC	0.08	0.2	[17]
Cd	51	200	19.1	BC	/	0.12	[18]
Bi-HgTe	60	178	24.2	BC	0.39	0.06	[19]
Ge-Sb	68	174	27	BC	/	0.15	[20]
Ge-Bi-$AgBiTe_2$	60	190	25.8	BC	0.45	0.14	[21]
Ca-In	98	230	47	RL and BC	0.1 (Ca)	0.2(Ca)	[22]
$AgInTe_2$	97	107	31.4	RL and BC	/	0.105	[23]
In	50	161	21.3	RL	/	/	[24]
Zn	127	205	42	RL	0.43	/	[25]
Bi-Zn	112	205	36	RL	/	0.27	[26]
Pb-Zn	109	229	30.4	RL	0.145	0.3	[27]
V	/	/	/	RL	/	/	[28]
W	/	/	/	RL	/	/	[29]
Al-Sb - $AgBiTe_2$	106	190	28.11	RL and BC	0.271 (experim ent)	0.19	This work

Table S2. Atomic energy levels of s, p, d and f states of different atoms (M=Sn, Te, Al, Ga, In, Tl, Au, Pt, Zn, Cu, Cd, Be, Mn, Mg, Eu, Ca, Sr, Ra, Ba, Li, Na, As, Bi, Sb, Ge, Si) (units, eV) are calculated by using the all-electron full-potential method[30].

Atom	Valence state	s-states	p-states	d-states	f-states
Sn	$5s^2 5p^2$	-10.91	-3.97		
Te	$5s^2 5p^4$	-15.45	-6.12		
Al	$3s^2 3p^1$	-8.04	-3.02		
Ga	$4s^2 4p^1$	-9.17	-2.92		
In	$5s^2 5p^1$	-8.33	-2.90		
Tl	$6s^2 6p^1$	-9.36	-3.09		
Au	$6s^1 5d^{10}$	-6.10		-6.43	
Pt	$6s^1 5d^9$	-6.09		-5.97	
Zn	$4s^2 3d^{10}$	-5.97		-9.99	
Cu	$4s^2 3d^9$	-5.89		-9.87	
Cd	$5s^2 4d^{10}$	-5.66		-11.46	
Be	$1s^2 2s^2$	-5.60			
Mn	$3d^5 4s^2$	-5.5		-8.70	
Ag	$5s^1$	-4.73			
Yb	$6s^2 5d^1 4f^{13}$	-4.70		-2.21	-9.14
Mg	$3s^2$	-4.70			
Eu	$6s^2 5d^1 4f^6$	-4.20		-3.16	-9.48
Ca	$4s^2$	-3.70			
Sr	$5s^2$	-3.50			
Ra	$7s^2$	-3.30			
Ba	$6s^2$	-3.23			
Li	$1s^2 2s^1$	-3.22			
Na	$2p^6 3s^1$	-3.04	-28.52		
As	$4s^2 4p^3$	-15.20	-5.83		
Bi	$6s^2 6p^3$	-14.05	-3.96		
Sb	$5s^2 5p^3$	-13.05	-5.27		
Ge	$4s^2 4p^2$	-12.20	-4.34		
Si	$3s^2 3p^2$	-11.37	-4.60		

Table S3. Bandgap of $Sn_{1.03-x-y}Al_xSb_yTe$–z%$AgBiTe_2$ samples measured by UV–Vis–NIR absorption spectrum.

$Sn_{1.03-x-y}Al_xSb_yTe$–z%$AgBiTe_2$ samples	Bandgap (eV)
x=y=z=0	0.228
x=0.02, y=0.04, z=0	0.298
x=0.08, y=0.015, z=0	0.283
x=0.08, y=0.015, z=2	0.279
x=0.08, y=0.015, z=4	0.271
x=0.08, y=0.015, z=6	0.243

Even though pristine $Sn_{1.03}Te$ has a smaller band gap compared to other samples, it does not show bipolar conduction until 873 K (Fig. 4 in the manuscript) due to its high intrinsic carrier concentration. Doping Sb in SnTe can slightly increase the bandgap, as has been explained by Tan et al.[31]. On the contrary, doping Al in SnTe should decrease the bandgap due to the increased population of occupied anti-bonding states which lift the L point. As a result, the bandgap first increases from SnTe to the sample x=0.02, y=0.04, z=0 and then slightly decreases to the sample x=0.08, y=0.015, z=0 due to the co-doping of Al and Sb. The band gap further decreases with increasing the alloying content of $AgBiTe_2$. This phenomenon has also been observed by Tan et al.[32], which can be attributed to two reasons. First, the bandgap of $AgBiTe_2$ (~0.16 eV) is smaller than that of SnTe and Al-Sb co-doped SnTe. Second, the lattice constant decreases with increasing the content of $AgBiTe_2$. This will enlarge the band dispersion and thus shrink the bandgap.

Table S4. *ZT* of lead-free thermoelectric materials

Thermoelectric materials	T (K) (ZT_{max})	ZT_{max}	ZT_{ave}
GeTe	700	2.5[33]	1.36 (400-750 K)
Mg_3Sb_2	700	1.4[34]	1.31 (400-700 K)
SnS_2	800	0.8[35]	0.43 (400-800 K)
MnTe	823	1.3[36]	0.6 (400-873 K)
BiCuSeO	923	1.1[37]	0.53 (400-873 K)
SiGe	1073	1.84[38]	0.91 (400-873 K)

Table S5. Densities of all the samples investigated in this study.

Samples	Experimental density (g cm^{-3})	Theoretical density (g cm^{-3})	Relative ratio (%)
SnTe	6.36	6.46	98.45
$Sn_{1.03}Te$	6.290	6.403	98.23
$Sn_{0.97}Al_{0.02}Sb_{0.04}Te$	6.172	6.342	97.32
$Sn_{0.91}Al_{0.04}Sb_{0.08}Te$	6.076	6.281	96.74
$Sn_{0.85}Al_{0.06}Sb_{0.12}Te$	5.911	6.220	95.03
$Sn_{0.8}Al_{0.08}Sb_{0.15}Te$	5.792	6.156	94.09
$Sn_{0.75}Al_{0.1}Sb_{0.18}Te$	5.749	6.092	94.37
$Sn_{0.8}Al_{0.08}Sb_{0.15}Te$-2%$AgBiTe_2$	5.864	6.191	94.72
$Sn_{0.8}Al_{0.08}Sb_{0.15}Te$-4%$AgBiTe_2$	5.875	6.225	94.38
$Sn_{0.8}Al_{0.08}Sb_{0.15}Te$-6%$AgBiTe_2$	5.885	6.257	94.05

Table S6. Thermoelectric parameters of n-type Bi_2Te_3 samples composed in the SnTe-based thermoelectric module.

T (K)	σ（S cm^{-1}）	S (μVK^{-1})	K (W$m^{-1}K^{-1}$)	ZT
300.35	1145.4	-195.32	1.4694	0.89313
348.69	868.57	-210.29	1.40584	0.95269
397.15	685.98	-215.44	1.47356	0.85814
445.78	568.53	-205.74	1.71309	0.62625
494.48	502.29	-176.84	2.10237	0.36946

Table S7. Parameters used for the modified Williamson-Hall model.

Parameters	Description	Value	Ref
λ	Wavelength of the synchrotron X-ray (Å)	1.5418	-
b	Magnitude of Burgers vector (m)	4.441×10^{-10}	-
$\bar{C}_{h00}$	Corresponding to the *h00* reflection determined by elastic modulus	0.186	39
C_{11}		115.15	
C_{12}	Elastic modulus (GPa)	4.70	40
C_{44}		21.37	
O	Non		

Table S8. Values of θ, FWHM, *hkl* and dislocation density in $Sn_{1.03-x-y}Al_xSb_yTe$-4%$AgBiTe_2$.

Parameters	Values			
θ(°)	14.244815	20.34283	25.18159	33.29579
(*hkl*)	(200)	(220)	(222)	(422)
FWHM (°)	0.27055	0.42488	0.48442	0.58772

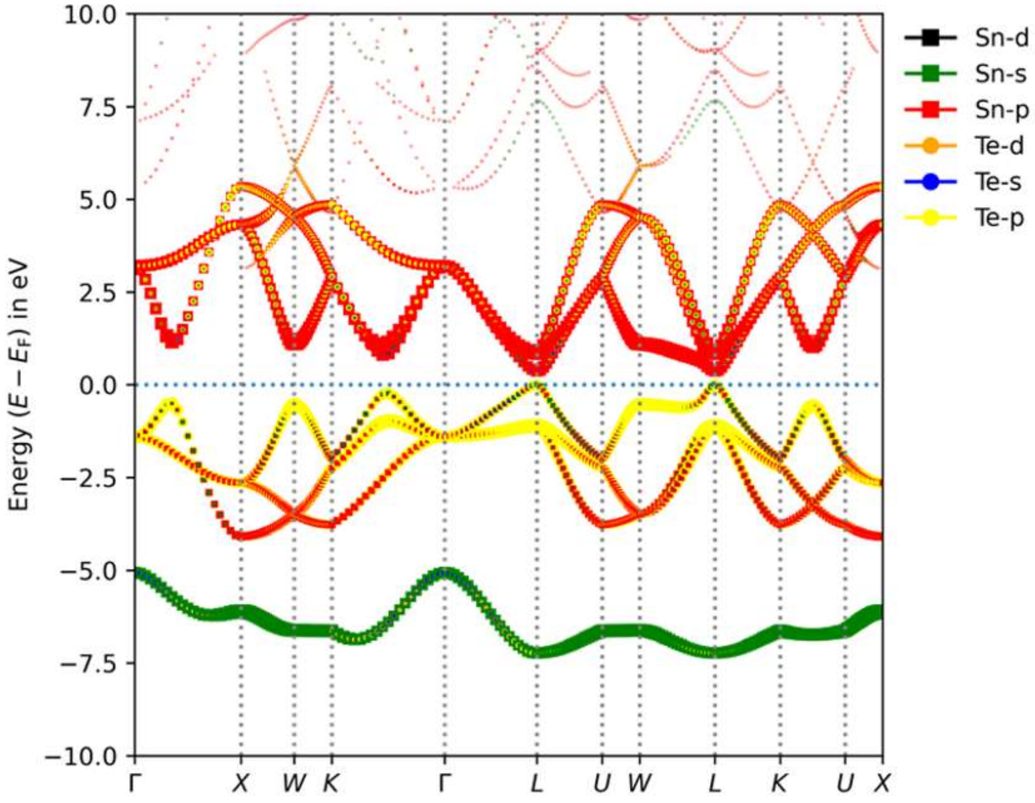

Figure S1. Orbital projected electronic band structuers (fat bands) of SnTe.

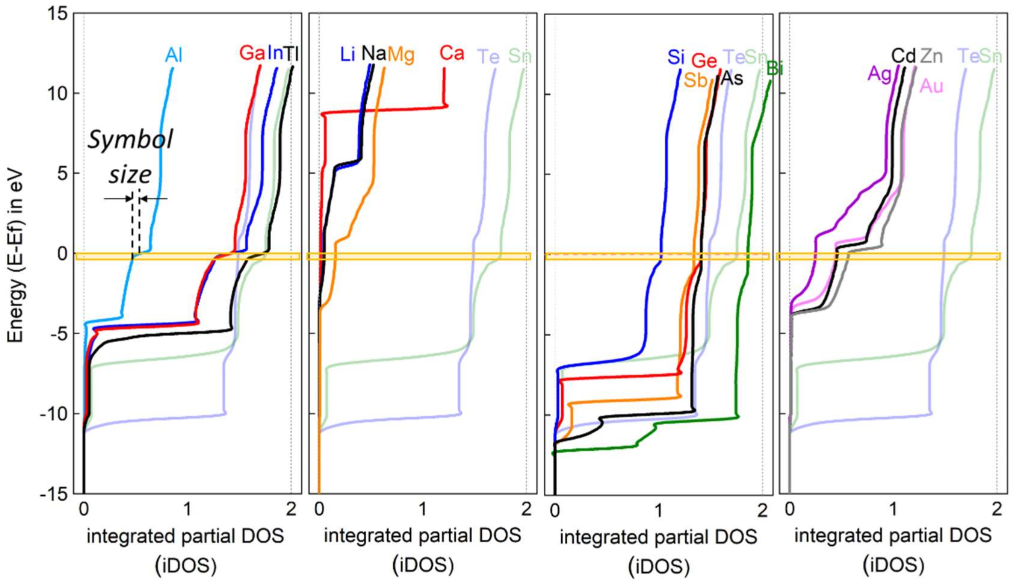

Figure S2. Integrated partial density-of-states (iDOS) for SnTe with various dopants. The symbol size in Figure 2 of the main manuscript scales to the difference between the iDOS at 0 eV and -0.1 eV.

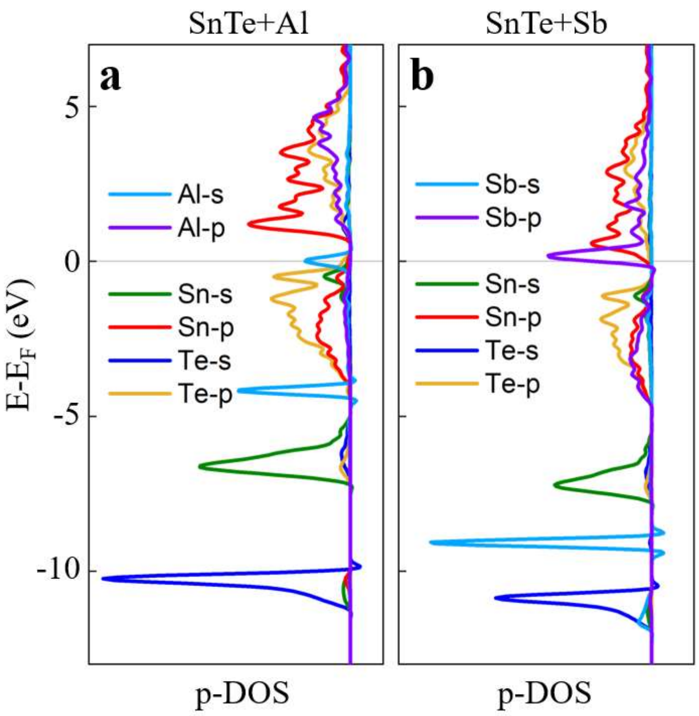

Figure S3. (a) Partial DOS of Al-doped SnTe, showing a ‘hump’ at the Fermi level contributed by the Al-s state. (b) Partial DOS of Sb-doped SnTe, where no DOS ‘hump’ from the dopant states is observed due to the large energy difference between Sb-s and Te-p states. On the contrary, the ‘hump’ of the Sn-s state near the Fermi level is even decreased.

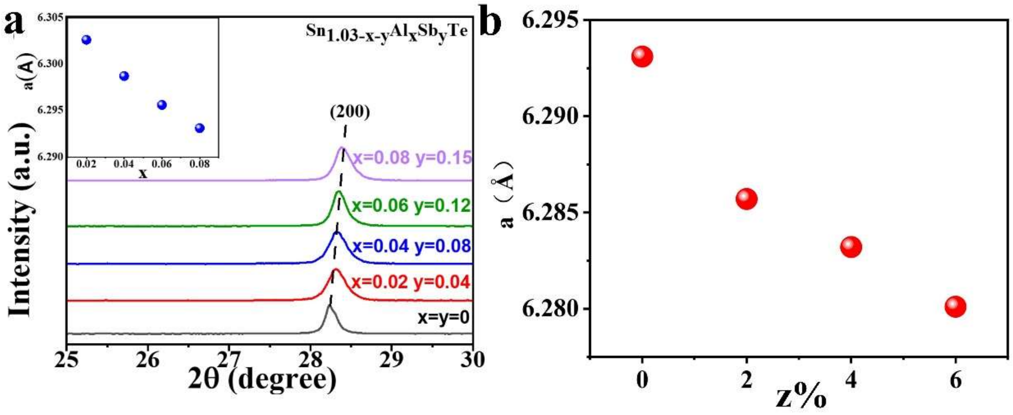

Figure S4. (a) Enlarged (200) diffraction peak of $Sn_{1.03-x-y}Al_xSb_yTe$ (x = 0, 0.02, 0.04, 0.06, 0.08, y=0, 0.04, 0.08, 0.12, 0.15), inset with lattice parameter;(b) Lattice parameter as a function of $AgBiTe_2$ fraction in $Sn_{0.8}Al_{0.08}Sb_{0.15}Te$–z%$AgBiTe_2$ (z=2, 4, 6).

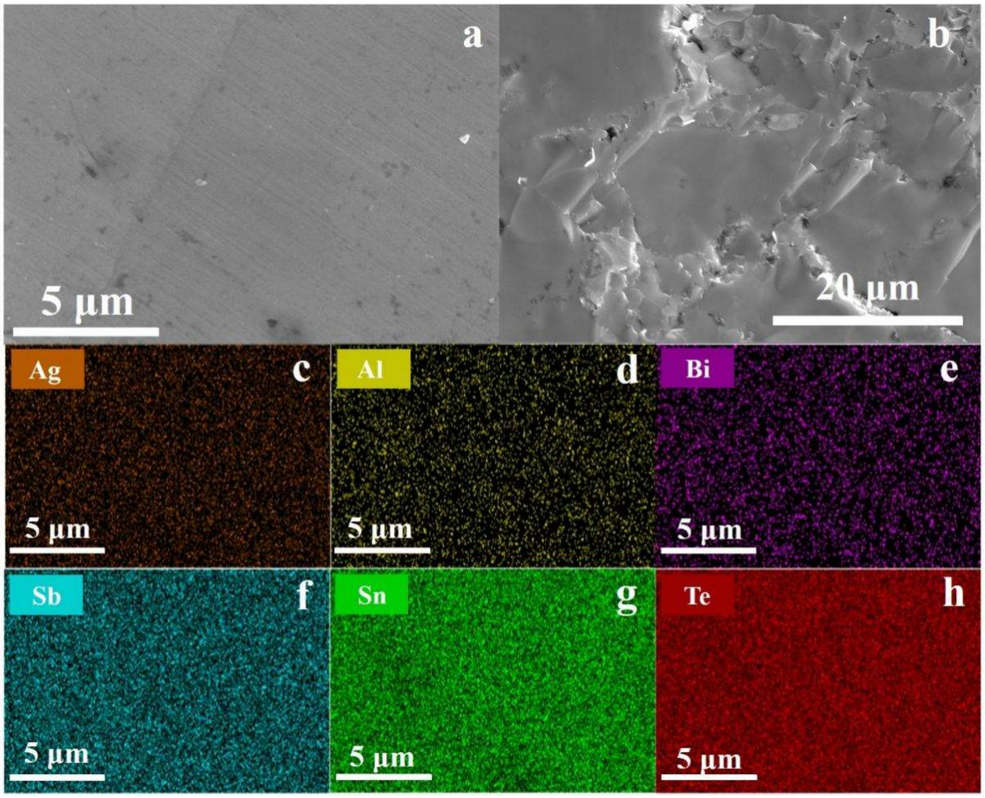

Figure S5. (a) Scanning electron microscope (SEM) image and fracture surface SEM image of $Sn_{0.80}Al_{0.08}Sb_{0.15}Te$–4%$AgBiTe_2$ (c-h) Elemental mapping of $Sn_{0.80}Al_{0.08}Sb_{0.15}Te$–4%$AgBiTe_2$ taken from the area in (a).

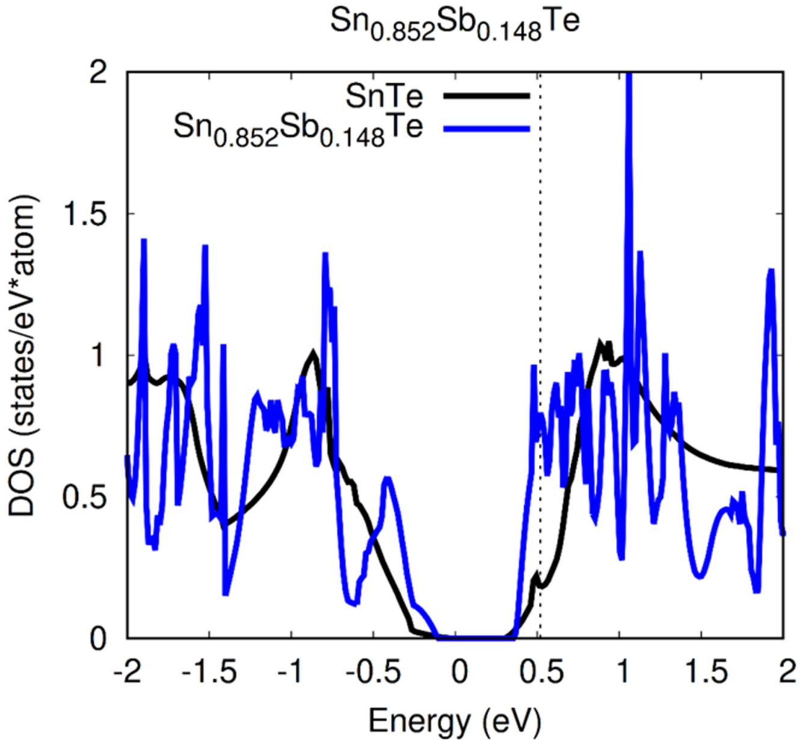

Figure S6. Electronic DOS of pristine SnTe and $Sn_{0.852}Sb_{0.148}Te$. The Fermi energy level of SnTe is set at 0 eV, while the dashed line indicates the Fermi energy level of Sb-doped SnTe.

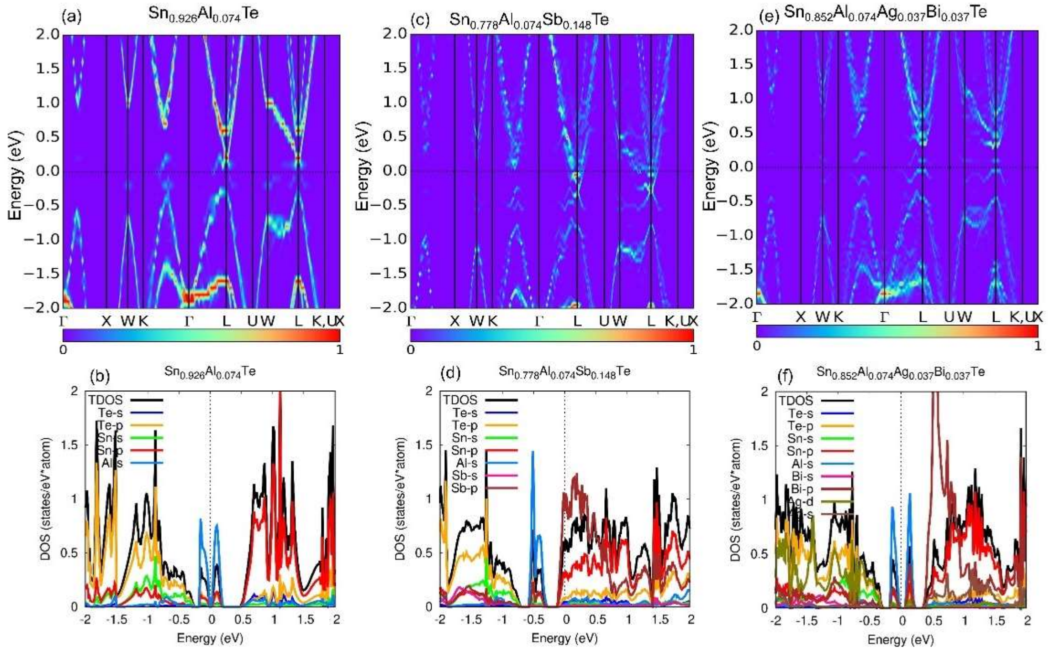

Figure S7. Electronic band structures of (a), (b) $Sn_{0.926}Al_{0.074}Te$; (c), (d) $Sn_{0.778}Al_{0.074}Sb_{0.148}Te$; and (e), (f) $Sn_{0.882}Al_{0.074}Ag_{0.037}Bi_{0.037}Te$.

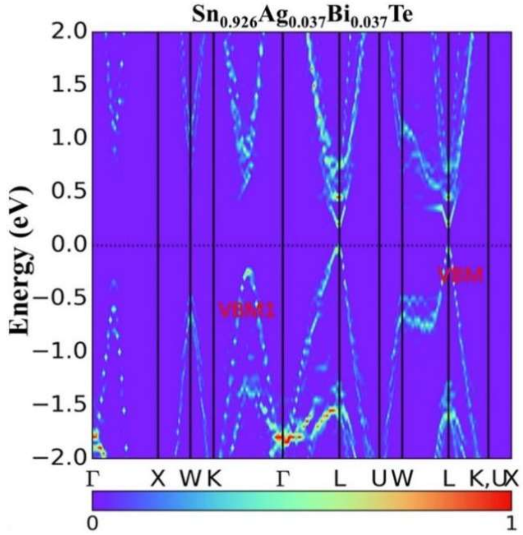

Figure S8. Electronic band structures of $Sn_{0.926}Ag_{0.037}Bi_{0.037}Te$. The scale bar is the magnitude of the spectral weight, which characterizes the probability of the primitive cell eigenstates contributing to a particular supercell eigenstate of the same energy.

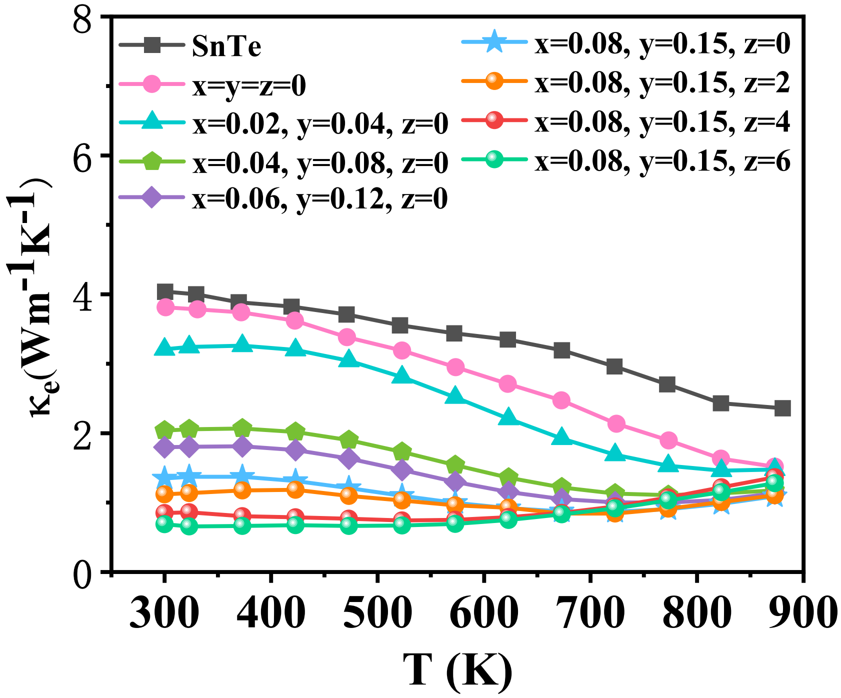

Figure S9. Electrical thermal conductivity of $Sn_{1.03-x-y}Al_xSb_yTe$–z%$AgBiTe_2$ as a function of temperature.

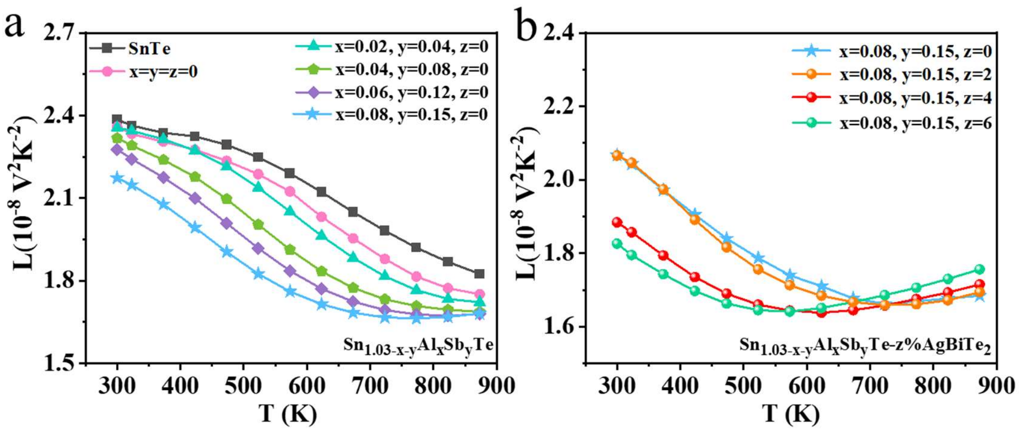

Figure S10. Lorenz number as a function of temperature for (a) $Sn_{1.03-x-y}Al_xSb_yTe$ and (b) $Sn_{0.8}Al_{0.08}Sb_{0.15}Te$–z%$AgBiTe_2$.

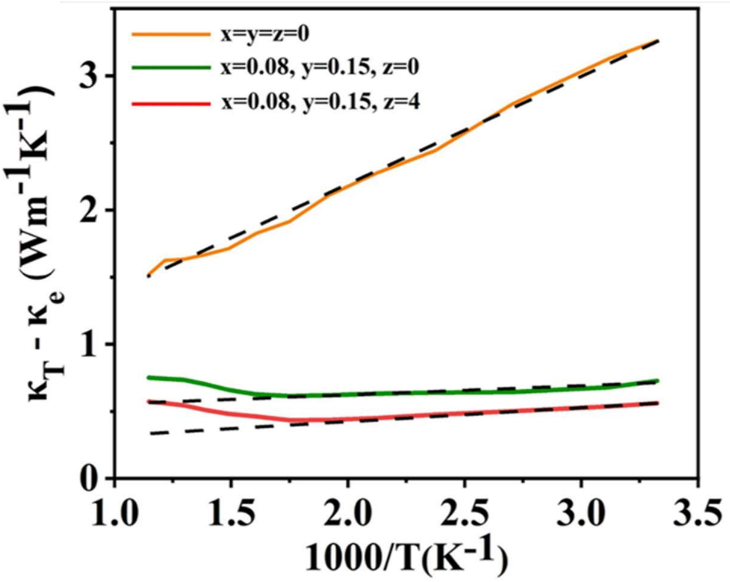

Figure S11. Total thermal conductivity minus electronic thermal conductivity κ_T - κ_L as a function of 1000/T of $Sn_{1.03-x-y}Al_xSb_yTe$-z%$AgBiTe_2$.

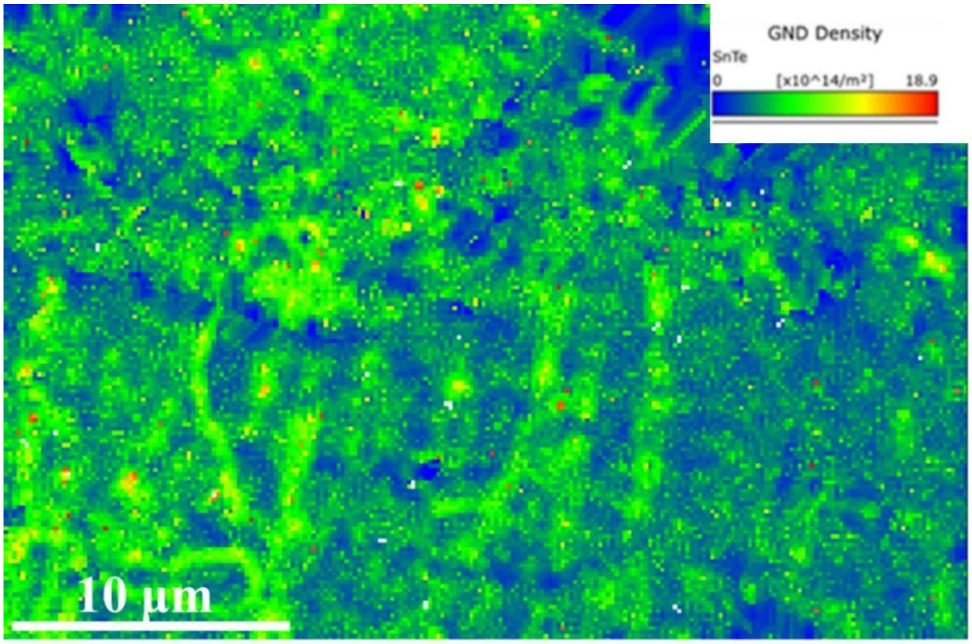

Figure S12. Geometrically necessary dislocation results of Scanning Electron Backscatter Diffraction for $Sn_{0.8}Al_{0.08}Sb_{0.15}Te$–4%$AgBiTe_2$.

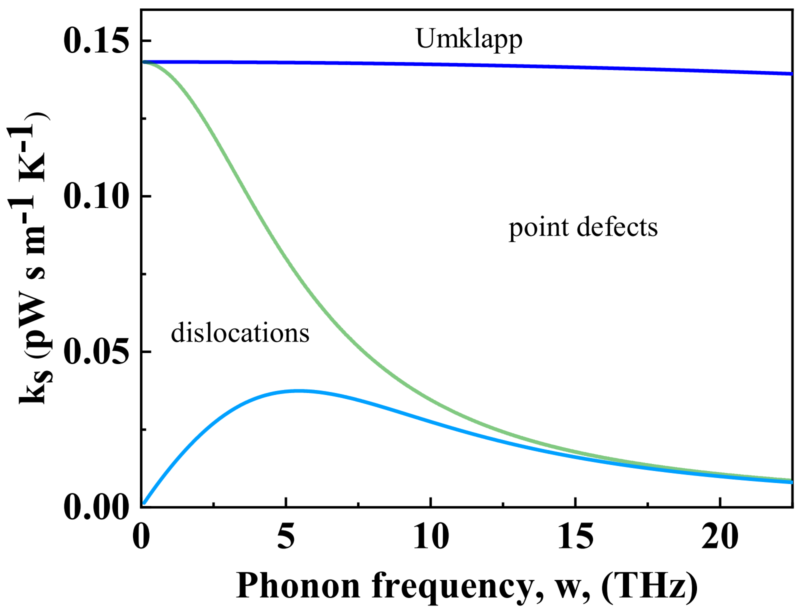

Figure S13. Spectral lattice thermal conductivity showing the frequency dependence of phonon scattering by different sources. Here, point defects (PD) mainly scatter high-frequency phonons while dislocations (DS) scatter low- to mid-frequency phonons.

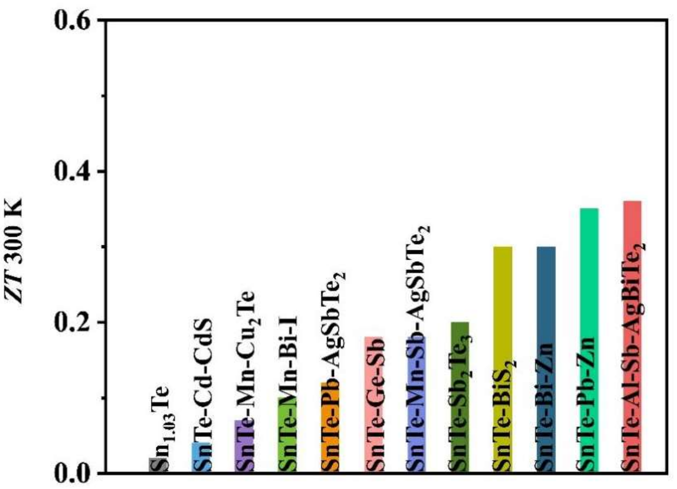

Figure S14. Comparison of *ZT* (300 K) of $Sn_{0.8}Al_{0.08}Sb_{0.15}Te$–4%$AgBiTe_2$ with other reported SnTe systems[18, 20, 26, 27, 41-46].

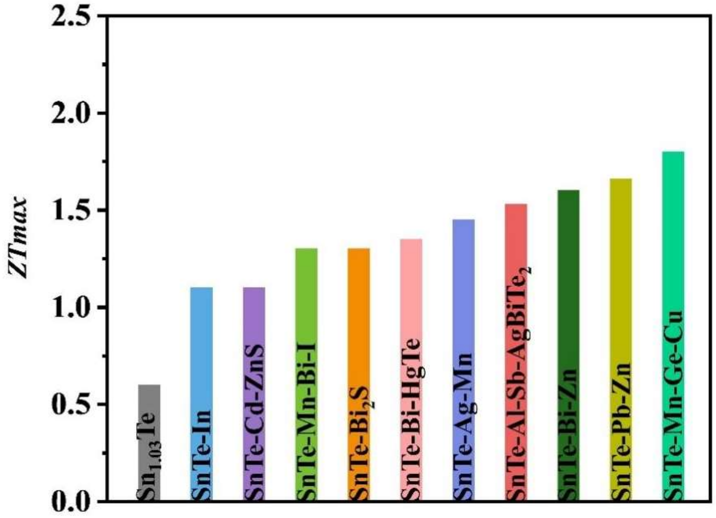

Figure S15. Comparison of ZT_{max} of $Sn_{0.8}Al_{0.08}Sb_{0.15}Te$–4%$AgBiTe_2$ with other reported SnTe systems[18, 19, 24, 26, 27, 44, 46-48].

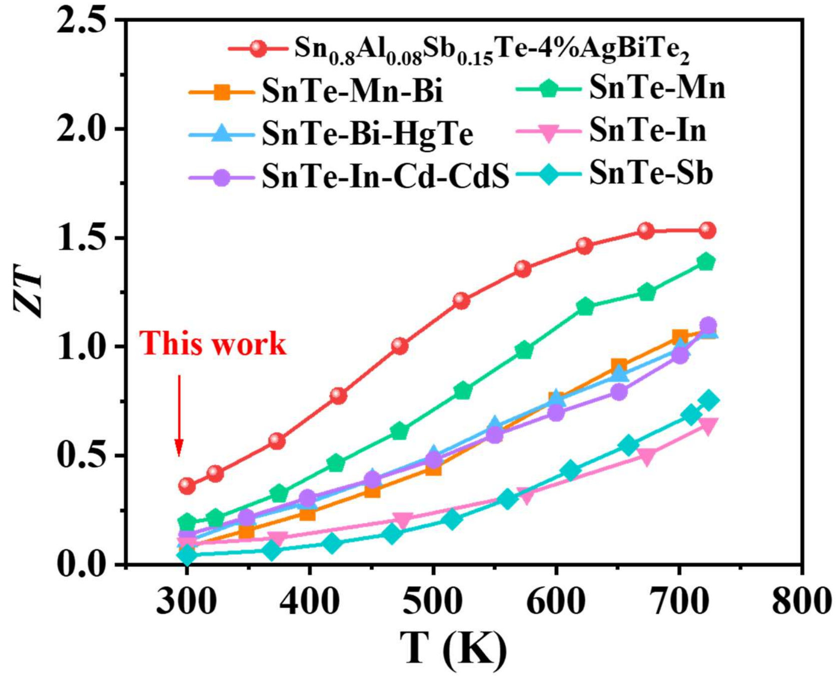

Figure S16. Comparisons of temperature-dependent *ZT* value at 300–723K in SnTe[19, 24, 41, 49-52].

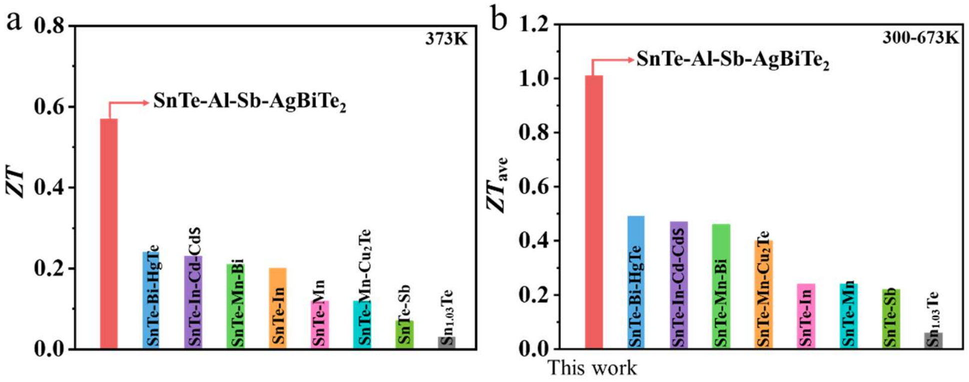

Figure S17. Comparisons of (a) *ZT* value at 373 K; (b) ZT_{ave} at 300-673K[19, 24, 41, 49-52].

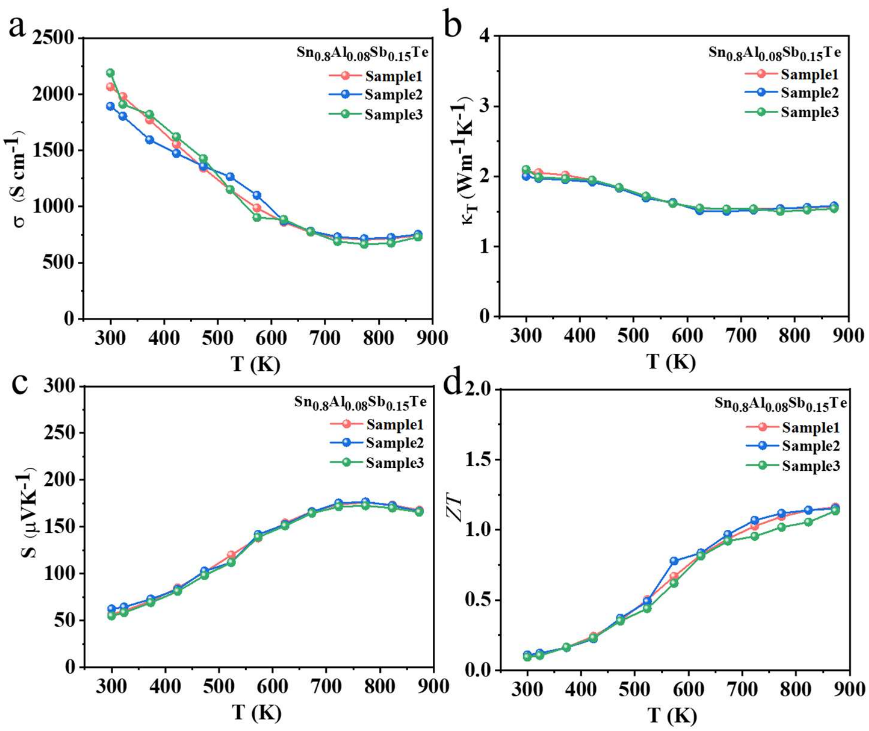

Figure S18. Reproducibility of thermoelectric properties as a function of temperature for $Sn_{0.8}Al_{0.08}Sb_{0.15}Te$: (a) Electrical conductivity; (b) Seebeck coefficient; (c) Thermal conductivity and (d) *ZT*.

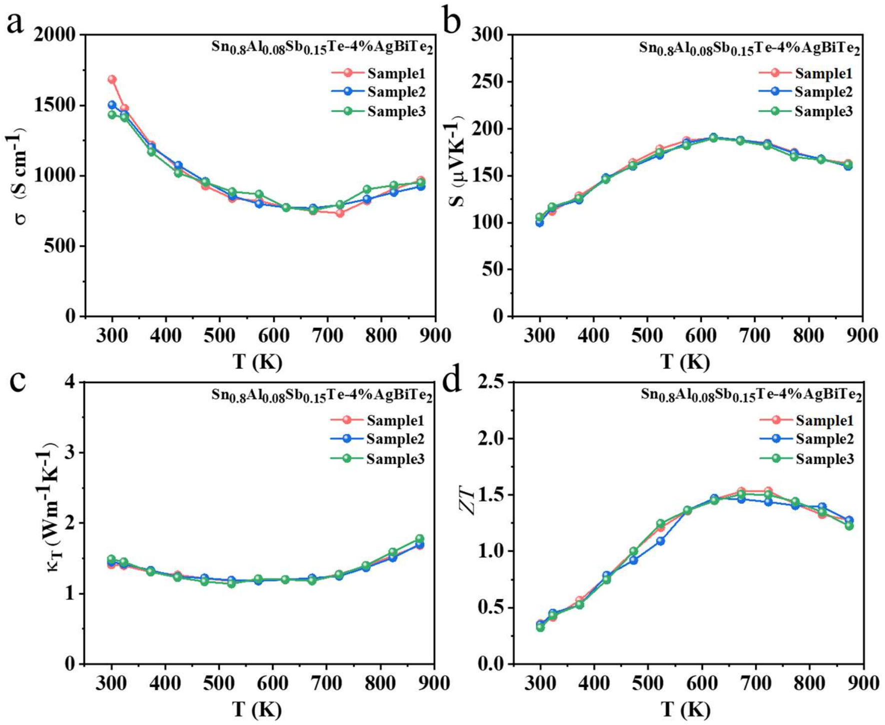

Figure S19. Reproducibility of thermoelectric properties as a function of temperature for $Sn_{0.8}Al_{0.08}Sb_{0.15}Te$–4%$AgBiTe_2$: (a) Electrical conductivity; (b) Seebeck coefficient; (c) Thermal conductivity and (d) *ZT*.

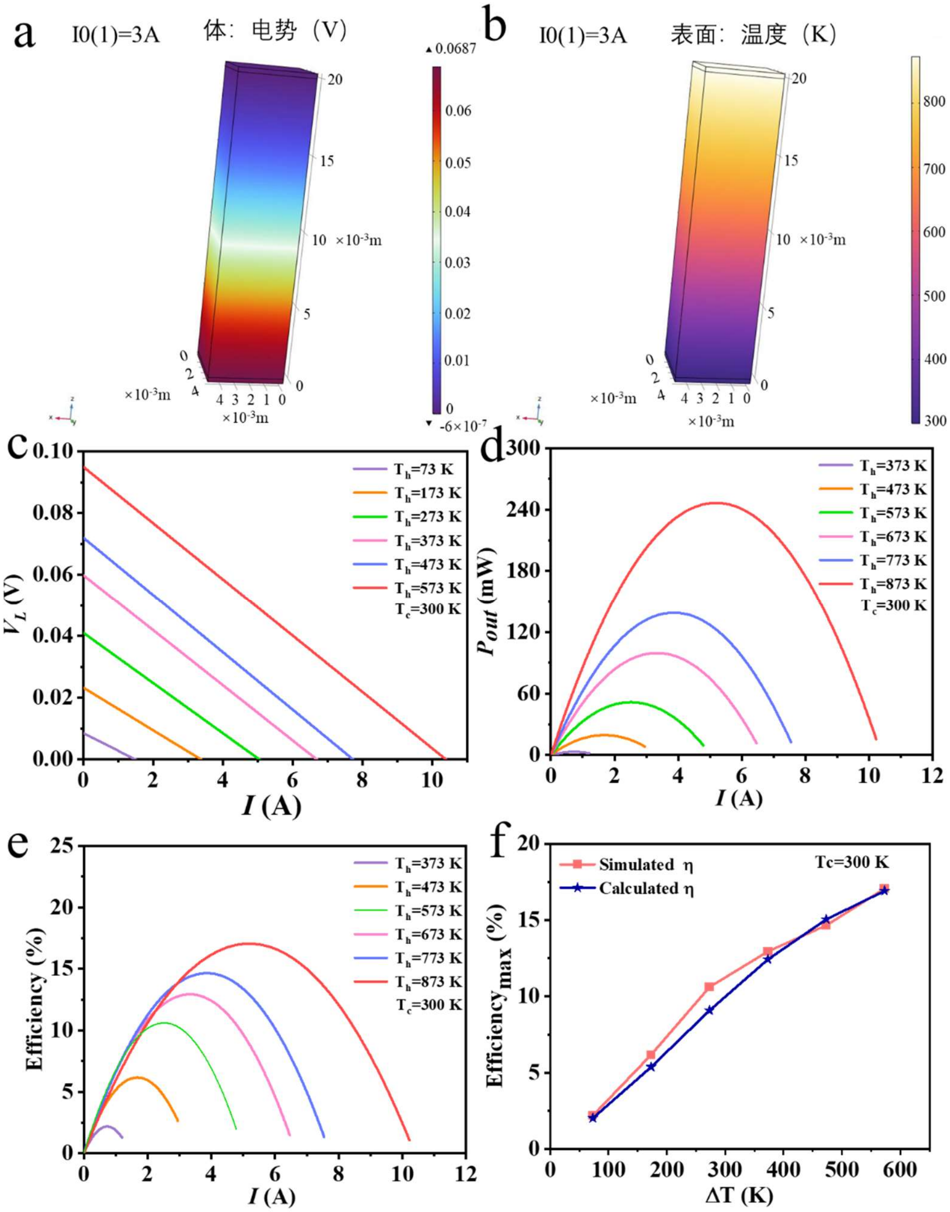

Figure S20. Theoretical values for conversion efficiency (η) of single-leg devices for $Sn_{0.8}Al_{0.08}Sb_{0.15}Te$–4%$AgBiTe_2$ based on COMSOL Multiphysics (a) temperature distribution of single-leg TE device; (b) corresponding electric potential distribution of the single-leg TE device; (c) Load voltage V_L versus current I; (d) output power P_{out} versus current I; (e) conversion efficiency versus current I, and (f) the η_{max} with different ΔT.

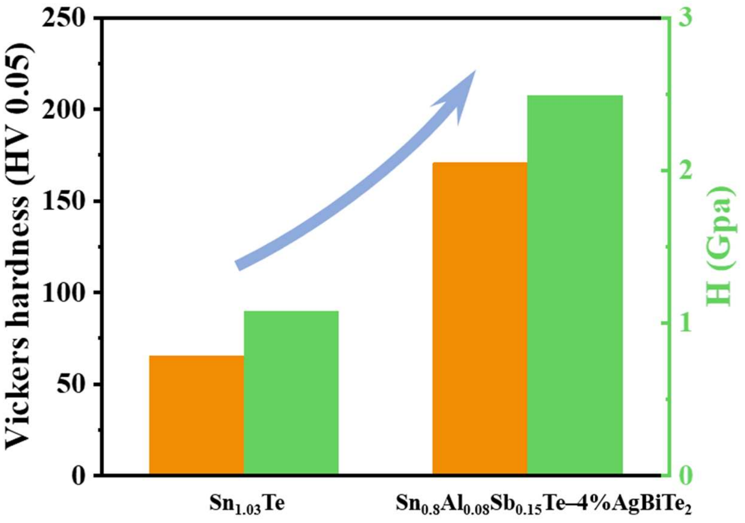

Figure S21. The Vickers microhardness and hardness (*H*) of $Sn_{1.03}Te$ and $Sn_{0.8}Al_{0.08}Sb_{0.15}Te$–4%$AgBiTe_2$.

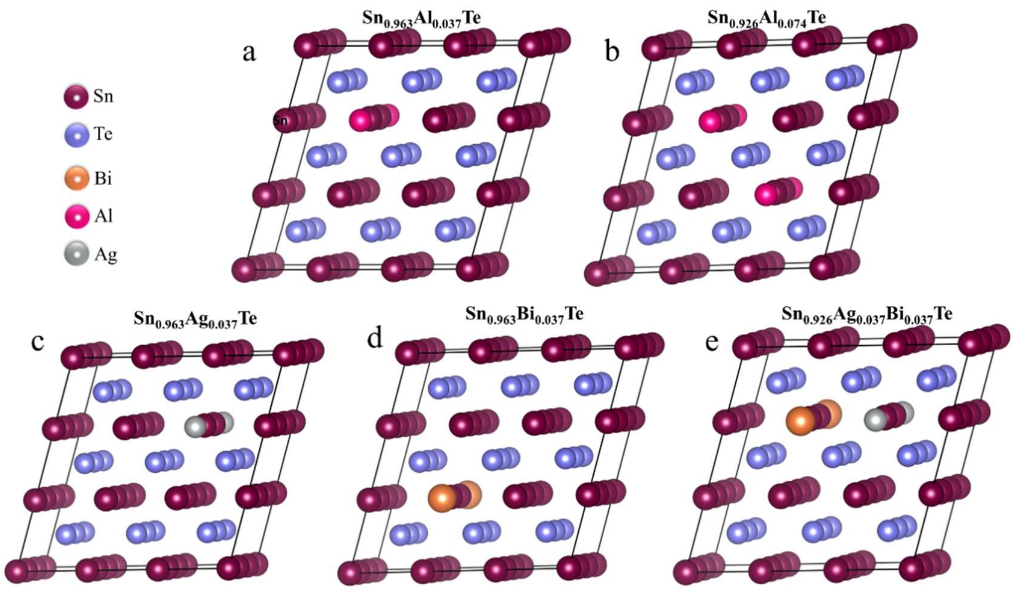

Figure S22. Crystal structures of (a) $Sn_{0.963}Al_{0.037}Te$, (b) $Sn_{0.926}Al_{0.074}Te$, (c) $Sn_{0.963}Ag_{0.037}Te$, (d) $Sn_{0.963}Bi_{0.037}Te$, (e) $Sn_{0.926}Ag_{0.037}Bi_{0.037}Te$.

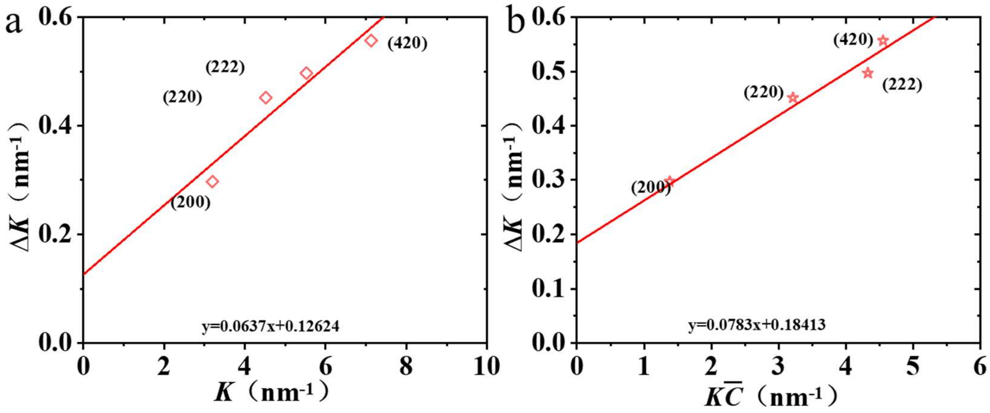

Figure S23. Estimation of dislocation density in $Sn_{0.8}Al_{0.08}Sb_{0.15}Te$–4%$AgBiTe_2$ by MWH method, (a) Plot of ΔK ~ K. (b) Plot of ΔK ~ $K\bar{C}^{1/2}$.

References

1. May, A. F., Toberer, E. S., Saramat, A. & Snyder, G. J. Characterization and analysis of thermoelectric transport in n-type $Ba_8Ga_{16-x}Ge_{30+x}$. *Phys. Rev B* **80**, (2009).
2. Zeier, W. G. et al. Influence of a Nano Phase Segregation on the Thermoelectric Properties of the p-Type Doped Stannite Compound $Cu_{2+x}Zn_{1-x}GeSe_4$. *J. Am. Chem. Soc.* **134**, 7147-7154, (2012).
3. Williamson, G. & Hall, W. X-Ray broadening from filed aluminium and tungsten. *Acta Metall.* **1**, 22-31 (1953).
4. Groma, I., Ungár, T. & Wilkens, M. Asymmetric X-Ray Line Broadening of Plastically Deformed Crystals. Part I: Theory. *J. Appl. Crystallogr.* **21**, 47-54 (1988).
5. Ungár, T., Groma, I. & Wilkens, M. Asymmetric X-ray line broadening of plastically deformed crystals. II. Evaluation procedure and application to [001]-Cu crystals. *J. Appl. Crystallogr.* **22**, 26-34 (1989).
6. Wilkens, M. The determination of density and distribution of dislocations in deformed single crystals from broadened X-ray diffraction profiles. *Phys. Status Solidi. A* **2**, 359-370 (1970).
7. Ungár, T., Ott, S., Sanders, P., Borbély, A. & Weertman, J. Dislocations, grain size and planar faults in nanostructured copper determined by high resolution X-ray diffraction and a new procedure of peak profile analysis. *Acta Metall.* **46**, 3693-3699 (1998).
8. Ungár, T. & Borbely, A. The effect of dislocation contrast on x-ray line broadening: A new approach to line profile analysis. *Appl. Phys. Lett.* **69**, 3173-3175 (1996).

9. Ungár, T., Gubicza, J., Borbely, A. & Ribarik, G. Crystallite size distribution and dislocation structure determined by diffraction profile analysis: principles and practical application to cubic and hexagonal crystals. *J. Appl. Crystallogr*, **34**, 298-310 (2001).

10. Wu, S. et al. Dislocation exhaustion and ultra-hardening of nanograined metals by phase transformation at grain boundaries. *Nat. Common.* **13**, 5468 (2022).

11. Xu, L. et al. Dense dislocations enable high-performance PbSe thermoelectric at low-medium temperatures. *Nat. Common.* **13**, 6449 (2022).

12. Carruthers, P. Scattering of phonons by elastic strain fields and the thermal resistance of dislocations. *Phys. Rev.* **114**, 995 (1959).

13. Zhu, Q. & Ren, Z. A double four-point probe method for reliable measurement of energy conversion efficiency of thermoelectric materials. *Energy* **191**, 116591-116599 (2020).

14. Hu, X., Yamamoto, A., Ohta, M. & Nishiate, H. Measurement and simulation of thermoelectric efficiency for single leg. *Rev. Sci. Instrum.* **86**, 526-532 (2015).

15. Al Rahal Al Orabi, R. et al. Band degeneracy, low thermal conductivity, and high thermoelectric figure of merit in SnTe-CaTe alloys. *Chem. Mater.* **28**, 376-384 (2016).

16. Banik, A., Shenoy, U. S., Anand, S., Waghmare, U. V. & Biswas, K. Mg alloying in SnTe facilitates valence band convergence and optimizes thermoelectric properties. *Chem. Mater.* **27**, 581-587 (2015).

17. Li. W, et al. Band and scattering tuning for high performance thermoelectric Sn_{1-}

$_{x}Mn_{x}Te$ alloys. *J. Materiomics* **1**, 307-315 (2015).

18. Tan, G. et al. High thermoelectric performance of p-type SnTe via a synergistic band engineering and nanostructuring approach. *J. Am. Chem. Soc.* **136**, 7006-7017 (2014).
19. Tan, G. et al. Extraordinary role of Hg in enhancing the thermoelectric performance of p-type SnTe. *Energy Environ. Sci.* **8**, 267-277 (2015).
20. Banik, A. et al. Engineering ferroelectric instability to achieve ultralow thermal conductivity and high thermoelectric performance in $Sn_{1-x}Ge_{x}Te$. *Energy Environ. Sci.* **12**, 589-595 (2019).
21. Nie, C. et al. Band Modification and Localized Lattice Engineering Leads to High Thermoelectric Performance in Ge and Bi Codoped SnTe-$AgBiTe_2$ Alloys. *Small* **19**, 2301298 (2023).
22. Bhat, D. K. & Shenoy, U S. Enhanced thermoelectric performance of bulk tin telluride: Synergistic effect of calcium and indium co-doping. *Mater. Today Phys.* **4**, 12-18 (2018).
23. Banik, A., Shenoy, U. S., Saha , S., Waghmare, U. V. & Biswas, K. High power factor and enhanced thermoelectric performance of SnTe-$AgInTe_2$: synergistic effect of resonance level and valence band convergence. *J. Am. Chem. Soc.* **138**, 13068-13075 (2016).
24. Zhang, Q. et al. High thermoelectric performance by resonant dopant indium in nanostructured SnTe. *P. Natl. Acad. Sci. USA* **110**, 13261-13266 (2013).
25. Bhat, D. & Shenoy, U. Zn: a versatile resonant dopant for SnTe thermoelectrics.

Mater. Today Phys. **11**, 100158 (2019).

26. Shenoy, U. S., & Bhat, D.K. Bi and Zn co-doped SnTe thermoelectrics: interplay of resonance levels and heavy hole band dominance leading to enhanced performance and a record high room temperature ZT. *J. Mater. Chem. C* **8**, 2036-2042 (2020).
27. Bhat, D. K. & Shenoy, U.S. SnTe thermoelectrics: dual step approach for enhanced performance. *J. Alloy. Compd.* **834**, 155181 (2020).
28. Shenoy, U. S. & Bhat, D. K. Vanadium: a protean dopant in SnTe for augmenting its thermoelectric performance. *ACS Sustainable Chem. Eng.* **9**, 13033-13038 (2021).
29. Shenoy, U. S., Goutham, K. & Bhat, D. K. Resonance states and hyperconvergence induced by tungsten doping in SnTe: Multiband transport leading to a propitious thermoelectric material. *J. Alloy. Compd.* **905**, 164146 (2022).
30. Schwarz, K. & Blaha, P. Solid state calculations using WIEN2k. *Comp. Mater. Sci.* **28**, 259-273 (2003).
31. Tan, X. et al. Designing band engineering for thermoelectrics starting from the periodic table of elements. *Mater. Today Phys.* **7**, 35-44 (2018).
32. Tan, G. et al. SnTe–$AgBiTe_2$ as an efficient thermoelectric material with low thermal conductivity. *J. Mater. Chem. A* **2**, 20849-20854 (2014).
33. Xing, T. et al. Ultralow lattice thermal conductivity and superhigh thermoelectric figure-of-merit in (Mg, Bi) co-doped GeTe. *Adv. Mater.* **33**, 2008773 (2021).
34. Wood, M., Kuo, J. J., Imasato, K. & Snyder, G. J. Improvement of low-temperature

ZT in a Mg_3Sb_2–Mg_3Bi_2 solid solution via Mg-vapor annealing. *Adv. Mater.* **31**, 1902337 (2019).

35. Zhan, S., Zheng, L., Xiao, Y. & Zhao, L-D. Phonon and carrier transport properties in low-cost and environmentally friendly SnS_2: a promising thermoelectric material. *Chem. Mater.* **32**, 10348-10356 (2020).

36. Zulkifal, S. et al. Multiple Valence Bands Convergence and Localized Lattice Engineering Lead to Superhigh Thermoelectric Figure of Merit in MnTe. *Adv. Sci.* 2206342 (2023).

37. Li, J. et al. A high thermoelectric figure of merit ZT> 1 in Ba heavily doped BiCuSeO oxyselenides. *Energy Environ. Sci* **5**, 8543-8547 (2012).

38. Basu, R. et al. Improved thermoelectric performance of hot pressed nanostructured n-type SiGe bulk alloys. *J. Mater. Chem. A* **2**, 6922-6930 (2014).

39. Ungár, T., Dragomir, I. & Borbély, A. The contrast factors of dislocations in cubic crystals: the dislocation model of strain anisotropy in practice. *J. Appl. Crystallogr.* **32**, 992-1002 (1999).

40. Muthumari, M., Manjula, M., Krishnaveni, S. & Pradheepa, K. Structural, electronic and mechanical properties of SnTe and selenium doped SnTe-Ab initio study. *Mater. Today: Proc.* **50**, 2741-2744 (2022).

41. Li, W. et al. Promoting SnTe as an eco-friendly solution for p-PbTe thermoelectric via band convergence and interstitial defects. *Adv. Mater.* **29**, 1605887 (2017).

42. Hong, T. et al. Band convergence and nanostructure modulations lead to high thermoelectric performance in $SnPb_{0.04}Te$-y% $AgSbTe_2$. *Mater. Today Phys.* **21**,

100505 (2021).

43. Xu, X. et al. Constructing van der Waals gaps in cubic-structured SnTe-based thermoelectric materials. *Energy Environ. Sci.* **13**, 5135-5142 (2020).

44. Chang, C. et al. Surface Functionalization of Surfactant-Free Particles: A Strategy to Tailor the Properties of Nanocomposites for Enhanced Thermoelectric Performance. *Angew. Chem. Int. Edit.* **61**, e202207002 (2022).

45. Liu, Y. et al. Improved solubility in metavalently bonded solid leads to band alignment, ultralow thermal conductivity, and high thermoelectric performance in SnTe. *Adv. Funct. Mater.* **32**, 2209980 (2022).

46. Hong, T. et al. Realizing ultrahigh room-temperature seebeck coefficient and thermoelectric properties in SnTe-based alloys through carrier modulation and band convergence. *Acta Mater.* **261**, 119412 (2023).

47. Tang, J. et al. Manipulation of Band Structure and Interstitial Defects for Improving Thermoelectric SnTe. *Adv. Funct. Mater.* **28**, 1803586 (2018).

48. Sarkar, D. et al. Highly Converged Valence Bands and Ultralow Lattice Thermal Conductivity for High-Performance SnTe Thermoelectrics. *Angew. Chem. Int. Edit.* **59**, 11115-11122 (2020).

49. Zhang, Q. et al. Improvement of thermoelectric properties of SnTe by Mn-Bi codoping. *Chem. Eng. J.* **421**, 2 (2021).

50. Tan, G. et al. Codoping in SnTe: enhancement of thermoelectric performance through synergy of resonance levels and band convergence. *J. Am. Chem. Soc.* **137**, 5100-5112 (2015).

51. Guo, F. et al. Ultrahigh thermoelectric performance in environmentally friendly SnTe achieved through stress-induced lotus-seedpod-like grain boundaries. *Adv. Funct. Mater.* **31**, 2101554 (2021).

52. Banik, A. et al. The origin of low thermal conductivity in $Sn_{1-x}Sb_xTe$: phonon scattering via layered intergrowth nanostructures. *Energy Environ. Sci.* **9**, 2011-2019 (2016).
